# In vitro performance of free and encapsulated bromelain

**DOI:** 10.1038/s41598-021-89376-0

**Published:** 2021-05-13

**Authors:** Janaína Artem Ataide, Letícia Caramori Cefali, Mariana Cecchetto Figueiredo, Lúcia Elaine de Oliveira Braga, Ana Lúcia Tasca Gois Ruiz, Mary Ann Foglio, Laura Oliveira-Nascimento, Priscila Gava Mazzola

**Affiliations:** 1 Graduate Program in Medical Sciences, Faculty of Medical Sciences, School of Medical Sciences, University of Campinas (UNICAMP), Tessália Vieira de Camargo Street, 126, Cidade Universitária “Zeferino Vaz”, Campinas, SP 13083-887 Brazil; 2 Graduate Program in Biosciences and Technology of Bioactive Products, Institute of Biology, University of Campinas (UNICAMP), Campinas, Brazil; 3 Graduate Program in Odontology, School of Odontology of Piracicaba, University of Campinas (UNICAMP), Piracicaba, Brazil; 4 Faculty of Pharmaceutical Sciences, University of Campinas (UNICAMP), Candido Portinari Street, 200, Cidade Universitária ″Zeferino Vaz″, Campinas, 13083-871 SP Brazil

**Keywords:** Drug delivery, Nanoparticles

## Abstract

For centuries, bromelain has been used to treat a range of ailments, even though its mechanism of action is not fully understood. Its therapeutic benefits include enzymatic debridement of the necrotic tissues of ulcers and burn wounds, besides anti-inflammatory, anti-tumor, and antioxidant properties. However, the protease is unstable and susceptible to self-hydrolysis over time. To overcome the stability issues of bromelain, a previous study formulated chitosan-bromelain nanoparticles (C-B-NP). We evaluated the optimized nanoformulation for in vitro antioxidant, cell antiproliferative activities and cell migration/proliferation in the scratch assay, comparing it with free bromelain. The antioxidant activity of free bromelain was concentration and time-dependent; after encapsulation, the activity level dropped, probably due to the slow release of protein from the nanoparticles. In vitro antiproliferative activity was observed in six tumor cell lines for free protein after 48 h of treatment (glioma, breast, ovarian, prostate, colon adenocarcinoma and chronic myeloid leukemia), but not for keratinocyte cells, enabling its use as an active topical treatment. In turn, C-B-NP only inhibited one cell line (chronic myeloid leukemia) and required higher concentrations for inhibition. After 144 h treatment of glioma cells with C-B-NP, growth inhibition was equivalent to that promoted by the free protein. This last result confirmed the delayed-release kinetics of the optimized formulation and bromelain integrity. Finally, a scratch assay with keratinocyte cells showed that C-B-NP achieved more than 90% wound retraction after 24 h, compared to no retraction with the free bromelain. Therefore, nanoencapsulation of bromelain with chitosan conferred physical protection, delayed release, and wound retraction activity to the formulation, properties that favor topical formulations with a modified release. In addition, the promising results with the glioma cell line point to further studies of C-B-NP for anti-tumor treatments.

## Introduction

*Ananas comosus* L., the common pineapple, has been used medically for centuries by native inhabitants of Central and South America to treat a range of ailments, mainly digestive disorders, and heal wounds^1,2^. Its medicinal properties are attributed to bromelain, a mixture of proteases and non-protease components, including other enzymes (phosphatases, glucosidases, peroxidases, and cellulases), glycoproteins, and carbohydrates^3^.

Several studies claim a wide range of medical applications for bromelain, such as inhibition of platelet aggregation, fibrinolysis, modulation of immune and inflammatory responses, antioxidant, antibacterial, and antifungal activities, enhanced absorption of other drugs, skin debridement, digestive aid, enhanced wound healing, and anti-carcinogenic effects^4–9^. Bromelain is sold as a nutritional supplement in health stores in the United States and Europe and is indicated for digestive health promotion, and as a wound treatment and anti-inflammatory agent^10,11^. Particular attention has been given to bromelain's antioxidant^12^ and antiproliferative activities^1–3,10,13–15^ and its wound healing properties^5,16,17^.

Drug delivery systems are changing the way of treating diseases, and nanotechnology has emerged in this field. These delivery systems aim to improve drug efficacy by enhancing the bioavailability of active drugs and reducing their adverse side-effects^13^. Furthermore, nanoparticulate systems offer other advantages as extensions of therapeutic drug effects at target sites and improve drug stability against chemical and enzymatic degradation^18^.

Therapeutic proteins present a particular challenge for drug therapy, primarily due to their immunogenicity and inflammatory potential, and physical and chemical degradation^19,20^. Therefore, nanotechnology to deliver protein drugs seems a plausible strategy for producing safe and effective therapeutic protein preparations and stabilizing protein drugs against denaturation by enzymatic digestion, thereby increasing their biopharmaceutical applications^21–24^.

Chitosan is a natural polysaccharide obtained from the deacetylation of chitin, which is very abundant. Chitosan presents favorable characteristics such as biocompatibility, biodegradability, and mucoadhesion, despite wound healing promotion, making it useful in the pharmaceutical industry, particularly in drug delivery systems^25^. Several different methods have been used to prepare chitosan nanoparticles, but ionotropic gelation is often favored, as it is a simple, mild, and controllable process^26^. In addition, bromelain has already been successfully encapsulated in chitosan nanoparticles by our group^27,28^.

Considering that nanoencapsulation modify proteins stability and other parameters, in this study, a series of in vitro assays were performed to investigate in vitro bromelain activity before and after the nanoencapsulation process. Our aim was to assess bromelain activity maintenance after encapsulation processes, comparing free and encapsulated bromelain performance in a series of in vitro activities, i.e., antioxidant activity, antiproliferative effects on tumor and non-tumor cell lines, and keratinocytes migration and proliferation in a scratch assay. Once chitosan has well known and reported properties, C-NP were also studied, investigating if observed effects could be attributed to bromelain or chitosan.

## Results

### Chitosan and chitosan-bromelain nanoparticles

**Figure 1:**
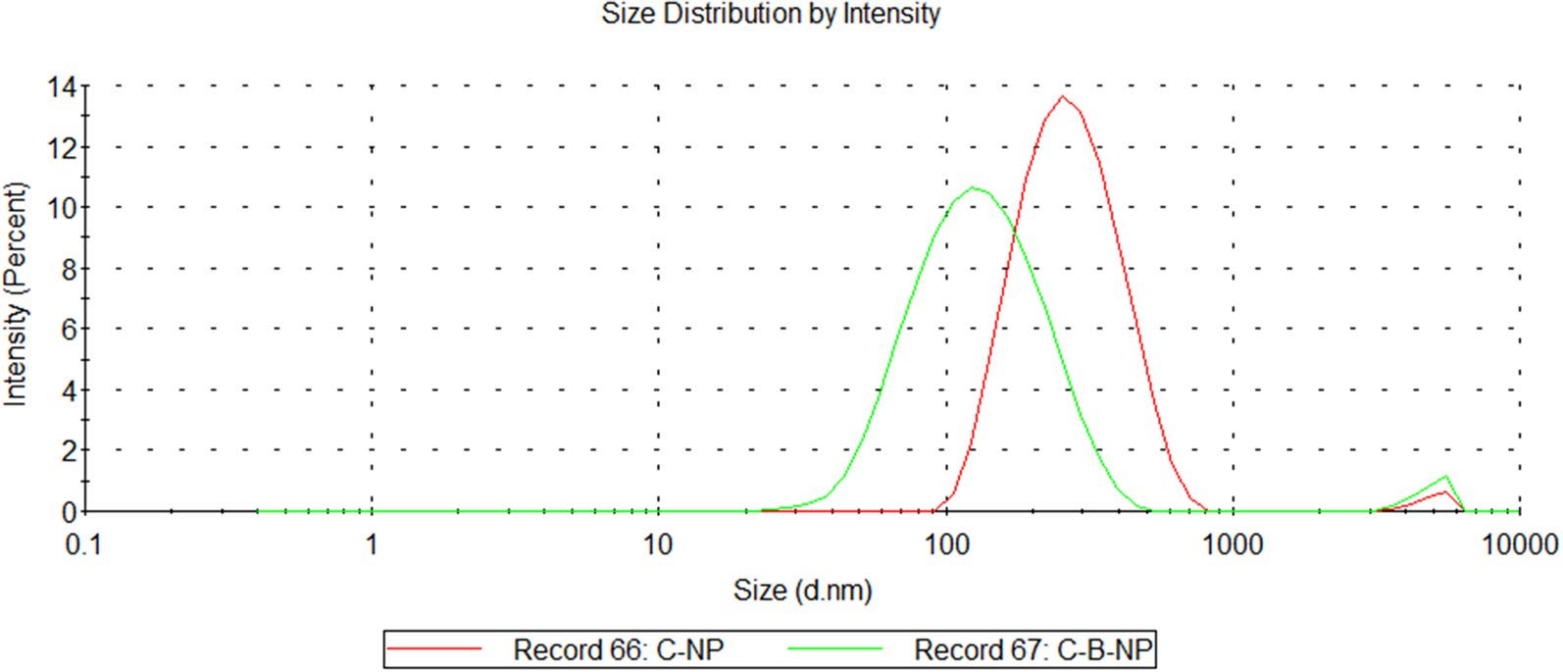
C-NP and C-B-NP intensity size distribution by dynamic light scattering. Result is presented as average data from three measurements and created by authors using Zetasizer software (version 8.01.4906, Malvern Panalytical).

Chitosan (C-NP) and chitosan-bromelain nanoparticles (C-B-NP) were successfully produced and characterized by dynamic light scattering (Fig. 1). Bromelain incorporation promoted a decrease in average particle size (118.9 ± 2.3) and a slight increase in the polydispersity index (0.260 ± 0.015) compared with empty chitosan nanoparticles (254.5 ± 1.4 and 0.222 ± 0.004, respectively). Zeta potential also changed with protein encapsulation, from 32.7 ± 1.2 (C-NP) to 21.1 ± 2.2 (C-B-NP).

### In vitro antioxidant activity

**Figure 2:**
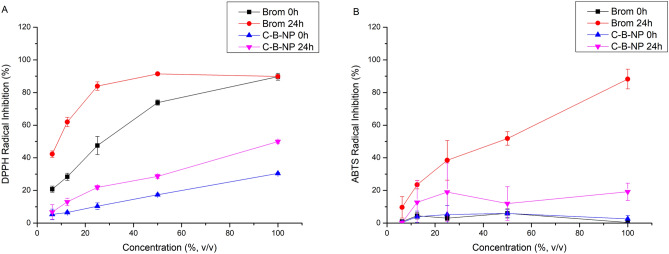
Antioxidant activity using (**A**) 2,2-diphenyl-1-picrylhydrazyl (DPPH) and (**B**) 2,2-azino-bis-3-ethylbenzothiazoline-6 sulfonic acid (ABTS) radicals. *Brom* bromelain solution, *Chi-Brom NP* chitosan-bromelain nanoparticles. Graphs were created by authors using average data with standard deviation (n = 3) from antioxidant assay, using Origin software (version 8.1.34.90, OriginLab Corporation).

In this study, the antioxidant activity of free bromelain, C-NP, and C-B-NP suspensions was evaluated using 2,2-diphenyl-1-picrylhydrazyl (DPPH, Fig. 2A) and 2,2-azino-bis-3-ethylbenzothiazoline-6 sulfonic acid (ABTS, Fig. 2B) radicals. Free bromelain showed higher antioxidant activity against DPPH than C-B-NP. C-B-NP showed up to 40% DPPH inhibition, equivalent to nearly half the free bromelain potency (89%). This pattern of decrease may be attributed to an inaccessible amount of bromelain. The inhibition with the encapsulated active increased up to 57% after 24 h, probably as an effect of free or surface-bound bromelain.

Concerning the ABTS radical, free bromelain showed significant antioxidant activity just after 24 h of incubation, which increased with increasing concentrations and reached 88% radical inhibition. The encapsulated protein inhibited ABTS radicals only in the 24 h sampling time, but with no concentration-dependent activity and up to 20% inhibition. Notably, 50% ABTS inhibition after 24 h required much more bromelain (50% v/v) than for DPPH inhibition (between 12.5 and 6.25%). Therefore, the amount of accessible protein in the encapsulated formulation may not be enough to elicit a concentration-dependent pattern.

### In vitro antiproliferative assay

**Figure 3:**
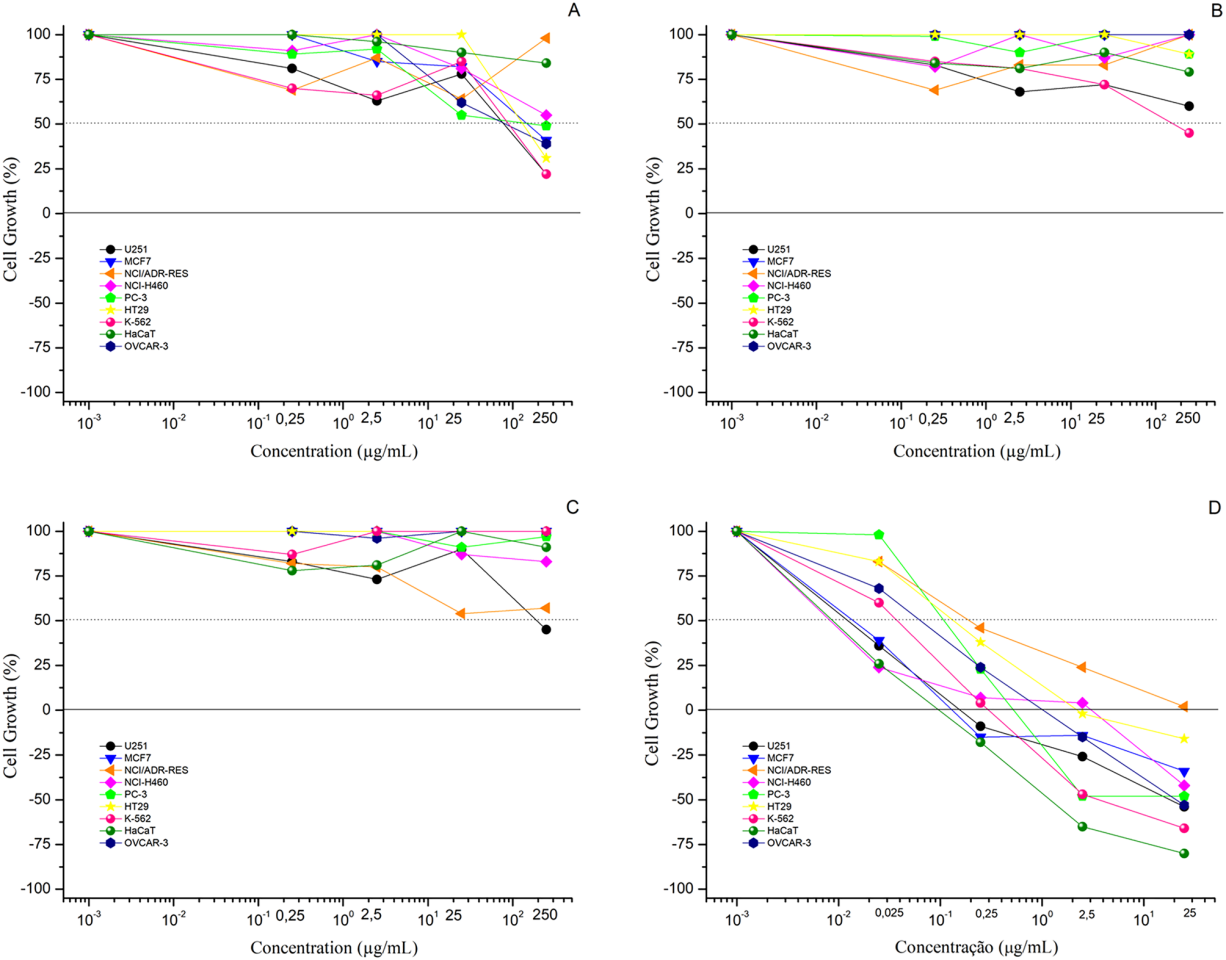
Antiproliferative activity of free bromelain solution (**A**), chitosan-bromelain nanoparticles (**B**), chitosan nanoparticles (**C**), and doxorubicin (**D**) after 48 h exposition. Graphs were created by authors using average data with standard deviation (n = 3) from antiproliferative assay, using Origin software (version 8.1.34.90, OriginLab Corporation).

Constituted by a mixture of proteases together with some other enzymes and proteins, there are some evidence of potential benefits of bromelain in cancer treatment^29^. In our study, we evaluate the antiproliferative effect of the free and nanoencapsulated commercial bromelain in a panel of eight human tumor cell lines of different histological or genetic origins and one human non-tumor cell line (Table 1, Fig. 3). Expressed as the concentration of bromelain required to inhibit 50% of cell growth (GI_50_), free bromelain showed weak antiproliferative effects against U251 (glioblastoma, GI_50_ = 44.9 mg/mL) and K562 (leukemia, GI_50_ = 60.7 mg/mL) cell lines. Moreover, all the three samples (free and nanoencapsulated bromelain besides empty chitosan nanoparticles) did not affect the proliferation of immortalized keratinocytes (HaCaT, GI_50_ > 250 mg/mL) (Table 1).

**Table 1:** In vitro antiproliferative effect, expressed as concentration required to 50% of cell growth inhibition (GI_50_, μg/mL) of doxorubicin (positive control), free bromelain solution, chitosan-bromelain nanoparticles (C-B-NP), and chitosan nanoparticles (C-NP) after 48 h-exposure.

Cell lines^b^	GI_50_^a^			
	Doxorubicin^c^	Bromelain^c^	C-B-NP^c^	C-NP^c^
U251	< 0.025	44.9*	> 250	250
MCF7	< 0.025	160.0 ± 63.2	> 250	> 250
OVCAR-03	0.057*	95.2 ± 43.9	> 250	> 250
NCI-ADR/RES	0.24 ± 0.06	> 250	> 250	> 250
NCI-H460	< 0.025	> 250	> 250	> 250
PC-3	0.23*	139.5 ± 129.7	> 250	> 250
HT29	0.13 ± 0.06	220.4 ± 1.3	> 250	> 250
K562	0.031*	60.7*	204.4 ± 124.5	> 250
HaCaT	< 0.025	> 250	> 250	> 250

^a^Results expressed as growth inhibition 50 (in μg/mL) followed by standard error, calculated by sigmoidal regression using Origin 8.0 software; *approximated value (experimental data did not converge, standard error higher than the calculated effective concentration).

^b^Human tumor cell lines: U251, glioblastoma; MCF-7, breast, adenocarcinoma; OVCAR-03, ovary, adenocarcinoma; NCI-ADR/RES, ovary, multi-drug resistant adenocarcinoma; NCI-H460, lung, non-small cell carcinoma; PC-3, prostate, adenocarcinoma; HT-29, colon, adenocarcinoma; K562, chronic myeloid leukemia. Human non-tumor cell line: HaCaT, immortalized keratinocytes.

^c^Samples: doxorubicin (chemotherapeutic drug; 0.025–25 μg/mL); bromelain (0.025–25 μg/mL, considering protein concentration); chitosan-bromelain nanoparticles (0.025–25 μg/mL, considering equivalent protein concentration of free bromelain); chitosan nanoparticles (0.025–25 μg/mL, considering equivalent amount of chitosan-bromelain nanoparticles).

**Figure 4:**
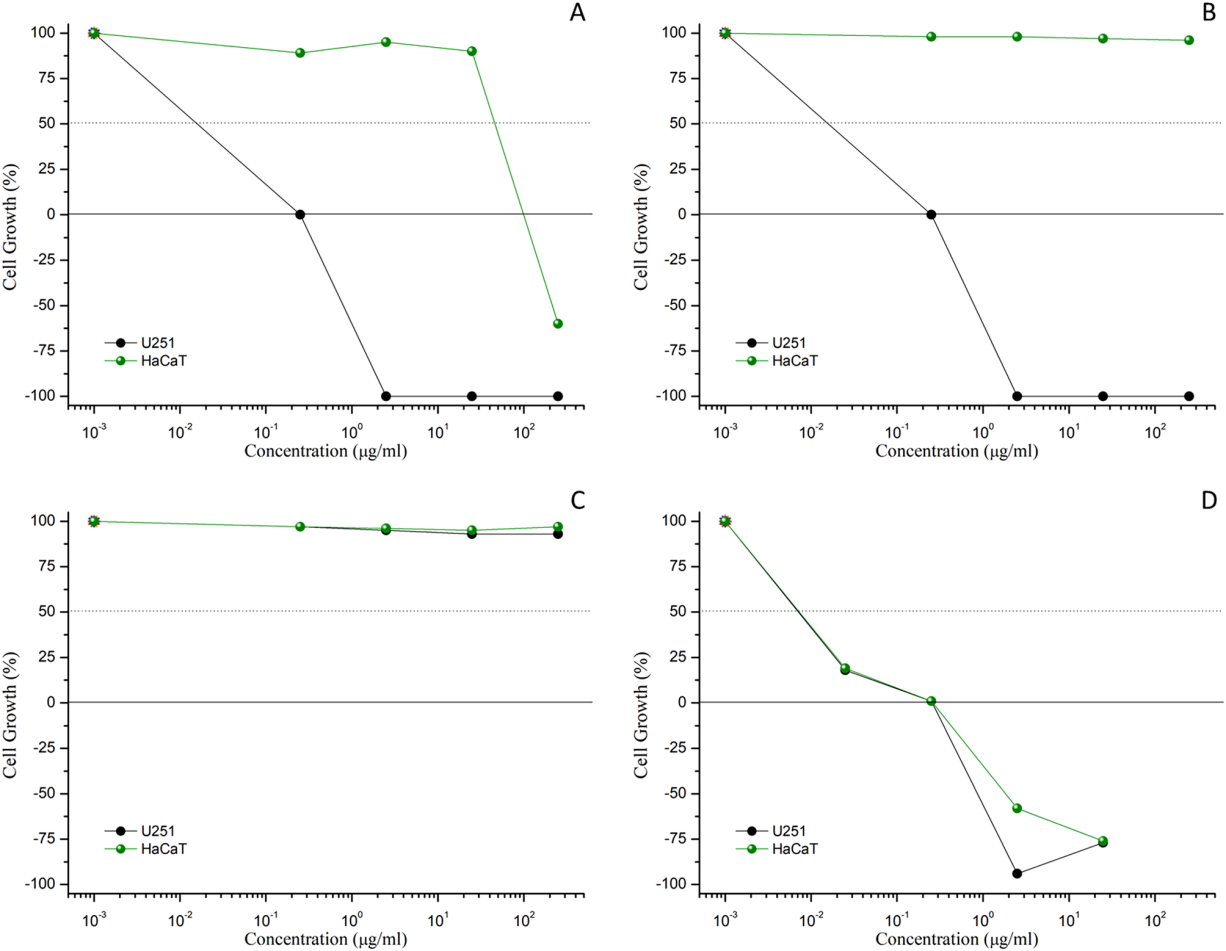
Antiproliferative activity of free bromelain solution (**A**), chitosan-bromelain nanoparticles (**B**), chitosan nanoparticles (**C**), and doxorubicin (**D**), after 144 h exposition. Data is presented as mean ± standard deviation; n = 3. Graphs were created by authors using average data with standard deviation (n = 3) from antiproliferative assay, using Origin software (version 8.1.34.90, OriginLab Corporation).

We also evaluated antiproliferative activity after 144 h treatment in U251 (glioblastoma) and HaCaT (immortalized keratinocytes) cell lines. The first one was the most sensitive cell line to bromelain in the first experiment for antiproliferative activity while the second cell line was representative of skin. As demonstrated in Table 2 and Fig. 4, the antiproliferative effects of free and nanoencapsulated bromelain against U251 cells were time-dependent. More, longer time exposure allowed the bromelain liberation from chitosan-nanoparticles resulting in similar antiproliferative effect. Further, as in the first antiproliferative experiment, empty chitosan-nanoparticles did not affect cell proliferation.

**Table 2:** In vitro time-dependent antiproliferative effect, expressed as concentration required to induce total cell growth inhibition (TGI, μg/mL), of doxorubicin (positive control), free bromelain solution, chitosan-bromelain nanoparticles (C-B-NP), and chitosan nanoparticles (C-NP).

Cell lines^b^	TGI^a^			
	U251		HaCaT	
	48^c^	144^c^	48^c^	144^c^
Doxorubicin^d^	0.26 ± 0.09	0.09 ± 0.08	0.09 ± 0.01	0.11 ± 0.06
Bromelain^d^	> 250	0.25	> 250	119.3 ± 21.4
C-B-NP^d^	> 250	0.25	> 250	> 250
C-NP^d^	> 250	> 250	> 250	> 250

^a^Results expressed as total growth inhibition (in μg/mL) followed by standard error, calculated by sigmoidal regression using Origin 8.0 software.

^b^Human tumor cell lines: U251, glioblastoma; Human non-tumor cell line: HaCaT, immortalized keratinocytes.

^c^Time exposure: 48 h and 144 h.

^d^Samples: doxorubicin (chemotherapeutic drug; 0.025–25 μg/mL); Bromelain (0.025–25 μg/mL, considering protein concentration); chitosan-bromelain nanoparticles (0.025–25 μg/mL, considering equivalent protein concentration of free bromelain); chitosan nanoparticles (0.025–25 μg/mL, considering equivalent amount of chitosan-bromelain nanoparticles).

### In vitro scratch assay

**Figure 5:**
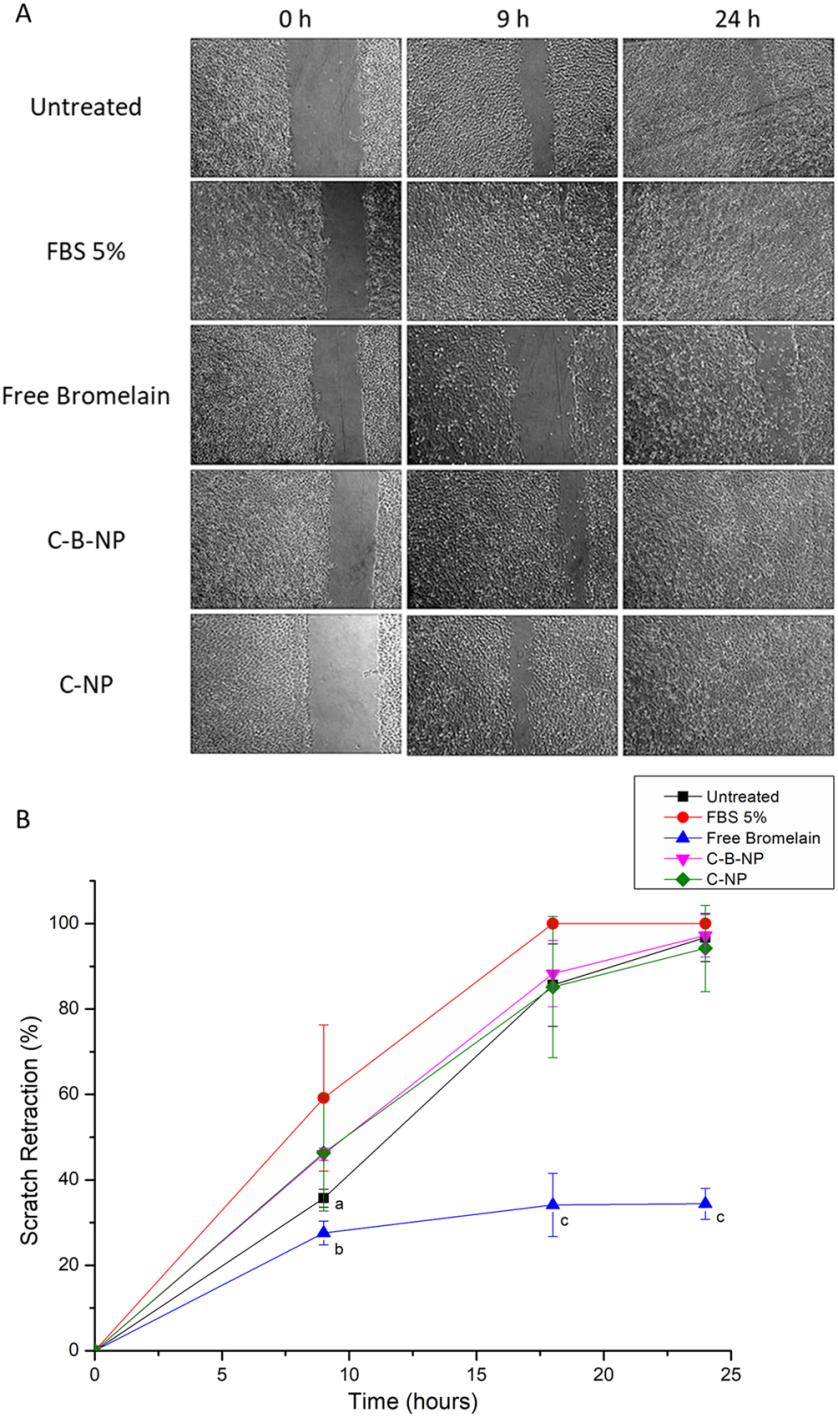
Representative micrographs of HaCaT (human non-tumor keratinocyte) cells treated with 250 µg/mL of controls and samples for 0, 9 and 18 h (A) and scratch retraction percentage of  controls and samples on scratch retraction during assay period (B). *FBS* fetal bovine serum, *Chi-Brom NPs* chitosan-bromelain nanoparticles, *Chi NPs* chitosan nanoparticles. Data is presented as mean ± standard deviation; n = 4. Letters represent statistical significance when comparing treatments in the same time point by Tukey’s test:  ^a^*p* < 0.05 when comparing scratch medium + FBS versus scratch medium;  ^b^*p* < 0.001 comparing scratch medium + FBS versus bromelain;  ^c^*p* < 0.001 comparing bromelain versus other treatments. Representative micrographs were chosen from micrographs taken by authors during in vitro scratch assay. Those micrographs were used to calculate the wound area and percentage of wound closure during assay, which was used by authors to create the graph.

Based on the observed antiproliferative effects on the immortalized keratinocytes, we evaluated the influence of free and nanoencapsulated bromelain, together with empty chitosan nanoparticles, in the HaCaT proliferation and migration using the in vitro scratch wound assay. Considering the three endpoint times evaluated (9, 18, and 24 h), fetal bovine serum supplementation accelerated the wound retraction after 9 and 18 h-exposure in comparison to untreated cells (Fig. 5). Regarding treatments, free bromelain reduced cell migration and proliferation (*p* < 0.001) at 18 and 24 h-endpoints compared to untreated cells.

Further, both empty chitosan nanoparticles (C-NP) and nanoencapsulated bromelain (C-B-NP) displayed a scratch retraction profile like that observed for untreated cells. Only at 18 h-endpoint, C-B-NP partially increased scratch retraction showing non-significant differences in comparison both to untreated and FBS 5%-treated cells.

## Discussion

C-B-NP characterization was in accordance with our previously reported data^27,28^. Decrease in zeta potential can be attributed to the bromelain surface's negative charge at pH 5.0^30^, which promotes electrostatic interaction between chitosan and bromelain, with a consequent decrease in free positive chitosan groups. Despite increase in PDI observed in C-B-NP compared with C-NP, both nanoparticles could be considered moderately polydisperse (PDI values between 0.1 and 0.4^31^). Encapsulation efficiency of C-B-NP was in accordance with previous reported results^27,28^. Protein release kinetics was not performed due to previous reported instability in relevant physiological medium overtime by other authors and our group^28^. In this same study^28^, TEM characterization of NP were performed and reported. Under TEM observation, particles presented spherical shape, with small particles aggregated in 100 nm agglomerates.

Antioxidant activity against DPPH and ABTS radicals was depended on the concentration and incubation time. Bromelain has been previously tested against DPPH and our results for the 30 min reaction (0 h) were similar to those previously reported, confirming test reproducibility^12,32^. The decrease of antioxidant activity of encapsulated bromelain corroborates with its high encapsulation rate and probable delayed release kinetics. Proteins can present antioxidant properties by physical interactions with metals, free radicals and other molecules, but also through chemical oxidation of their structure, besides other specific mechanisms^33^. The interaction bromelain-chitosan evidenced by changes in zeta potential probably dimished free binding sites that would otherwise promote antioxidant activity.

Different experiments have demonstrated that cytotoxic effects of bromelain nanoparticles were positive correlated with incubation time as increased time exposure resulted in more intense effect. Considering the chemical composition complexity of bromelain, there are some reports correlating the pharmacological efficacy as antitumor and/or immunogenic agents with the proteolytic activity of bromelain^6^.

The anticancer mechanism of action of bromelain have been investigated in different tumor cell lines. Bromelain increased reactive oxygen species and superoxide levels, leading to high autophagy induction in colorectal cancer cells. Elevation of apoptotic proteins amounts triggering caspase-dependent and independent apoptotic pathways were also observed in cells from human colon adenocarcinomas^34^. Reduction of CD44 surface marker expression, involved in tumor proliferation and migration, was reported after bromelain treatment on leukemia, melanoma and glioma cells^35,36^. This reduction was attributed to bromelain proteolytic activity, cleaving cells receptors and resulting in reduced cell invasion, migration, and adhesion in glioma cells^36^. Cleavage of transmembrane proteins may also interfere in intracellular signaling process^36,37^.

Although it did not affect the proliferation of immortalized keratinocytes after 48 h of exposure (antiproliferative activity test), free bromelain inhibited the migration and proliferation of this same keratinocyte strain in the scratch model. This can be attributed to the absence of fetal bovine serum (FBS). FBS contains different substances that act as inducers of cell migration and proliferation. Therefore the scratch experimental model was standardized with the reduction and/or total suppression of serum at least during the period of cells treatment with samples under study. A consequence of this withdrawal of FBS is the decrease in the concentration of proteins present in the culture medium. Thus, the proteolytic action of bromelain could be affecting cells more directly than in the condition of cultivation with medium supplemented with 5% FBS. A similar effect was reported by Schulz et al.^38^ when comparing the effect of a formulation containing bromelain on the viability of human fibroblasts and keratinocytes (primary cultures) in complete culture medium or in phosphate-buffered saline (PBS). The replacement of culture medium with PBS intensified the cytotoxic effect of bromelain on both cells evaluated.

Still, other studies have shown that different preparations of bromelain could induce cell cycle arresting in G0 phase (quiescence), which would explain the inhibition of cell proliferation^39^. These results reinforce the understanding that the healing effect of bromelain in wound care is mainly due to its debriding action, which removes cell debris and necrotic tissues in vivo, in accordance with other studies^6,38,40,41^.

Been a promisor biopolymer for many reasons, chitosan polymers have been described as a wound healing promotor by stimulating fibroblast proliferation and type IV collagen synthesis^32,42,43^. More, this pharmacological effect can be modulated by polymeric grade, the degree of N-deacetylation and presence of other ligands^42,44^. In our study, although chitosan nanoparticles did not increase cell migration or proliferation, they were able to avoid the inhibitory effect of free bromelain (Fig. 4).

## Conclusion

The data obtained in the in vitro models employed suggest that the nanoencapsulation system allowed the prolonged release of bromelain resulting in antioxidant and antiproliferative effects depending on the time of exposure. After 48 h, free bromelain produced antiproliferative effects against six tumor cell lines. Conversely, C-B-NP inhibited proliferation of only chronic myeloid leukemia cell line, and this effect required a higher concentration than that of free bromelain. After 144 h treatment, free bromelain and C-B-NP completely inhibited glioma cell growth, confirming bromelain integrity and delayed-release kinetics. During the antiproliferative assay, free and encapsulated bromelain did not inhibit keratinocyte cell growth, enabling their topical usage. Finally, in the scratch assay, C-B-NP promoted more than 90% wound retraction after 24 h, unlike free bromelain, which did not produce wound retraction. Chitosan used as wall material in nanoencapsulation also added wound retraction property to the final formulation. Therefore, nanoencapsulation of bromelain with chitosan conferred physical protection, delayed release, and wound retraction activity to the formulation, properties that favor topical formulations with a modified release. In addition, the promising results with the glioma cell line point to further studies of C-B-NP for anti-tumor treatments.

## Material and methods

### Materials

Bromelain extracted from pineapple stem (catalog number B4882), low molecular weight chitosan (catalog number 448869), azocasein (catalog number A2765) and Bradford reagent (catalog number B6916) were purchased from Sigma-Aldrich (Sao Paulo, Brazil). All other reagents were of analytical grade.

### Chitosan and chitosan-bromelain nanoparticles formulation and characterization

C-B-NP were produced by ionotropic gelation method as previously described^27,28^. Briefly, TPP solution (0.5 mg/mL in distilled water and filtered at 0.22 μm, 3 mL) was added dropwise to low molecular weight chitosan solution (2.5 mg/mL in 1% (v/v) acetic acid at pH 5.0, and filtered at 0.45 μm, 2 mL). Immediately after that, 1 mL of 0.22 µm filtered bromelain solution (10 mg/mL) or water was added and mixed under magnetic stirring (Fisatom, Mod 753E, Sao Paulo, Brazil) at 350 rpm for 40 min to afford chitosan-bromelain (C-B-NP) or chitosan nanoparticles (C-NP), respectively.

Physicochemical parameters of average particle size, polydispersity index, and zeta potential were determined using a Zetasizer Nano ZS90 (Malvern Instruments, Malvern, UK) instrument without previous dilution.

### In vitro antioxidant activity

Antioxidant activity was assessed using 2,2-diphenyl-1-picrylhydrazyl (DPPH) and 2,2-azino-bis-3-ethylbenzothiazoline-6 sulfonic acid (ABTS)^45–47^. Five different sample concentrations were prepared by diluting the tested samples (bromelain solution, chitosan-bromelain, and chitosan nanoparticles) in distilled water to obtain final concentrations of 6.25%, 12.5%, 25%, 50%, and 100% (v/v). In general, 2.5 mL of sample was mixed with 2.5 mL of DPPH radical solution, incubated for 30 min and read at 531 nm. For the ABTS assay, 30 µL of the sample was mixed with 3 mL of ABTS radical solution, vortexed for 6 min, and read at 734 nm. After the first measurement, the samples were sealed and protected from light, and the absorbance was reread after 24 h. Water was used as the negative antioxidant control. C-NP present a small inhibition of DPPH and ABTS; thus, its absorbance was considered and deduced from C-B-NP absorbance. The free radical sequestering capacity was calculated in relation to the absorbance of the radical solution with water, as follows:

1 $$Inhibition \left( \% \right) = \frac{{Abs_{control} - Abs_{sample} }}{{Abs_{control} }} \times 100$$

### In vitro assays

#### Cell lines

A panel of eight human tumor cell lines were tested: U251 (glioma), MCF-7 (breast), OVCAR-03 (ovarian), NCI-ADR/RES (ovarian expressing phenotype of multiple drug resistance), NCI-H460 (lung, non-small cells), PC-3 (prostate), HT-29 (colon adenocarcinoma), and K-562 (chronic myeloid leukemia). These cells were kindly provided by the Frederick Cancer Research & Development Center (National Cancer Institute, Frederick, MA, USA). The human non-tumor cell line HaCat (keratinocyte) was provided by Dr. Ricardo Della Coletta (University of Campinas-UNICAMP, Brazil). For all experiments, all cell lines were used between passages 5 and 12.

Stock cultures were grown in 5 mL of RPMI-1640 supplemented with 5% fetal bovine serum (RPMI/FBS 5%) and a 1% penicillin: streptomycin mixture (1000 U/mL:1000 μg/mL) (complete medium) at 37 °C and 5% CO_2_. For the scratch assay, samples were diluted in RPMI-1640 supplemented with 0.2% fetal bovine serum and 1% penicillin–streptomycin mixture (1000 U/mL:1000 μg/mL) (scratch medium).

#### Sample preparation

Bromelain, C-B-NP and C-NP stock solutions (5 mg/mL) were prepared in water and then successively diluted with complete (antiproliferative assay) or scratch (scratch assay) media to achieve final concentrations of 0.25, 2.5, 25, and 250 μg/mL (antiproliferative assay) and 250 μg/mL (scratch assay). Doxorubicin (0.025, 0.25, 2.5, and 25 μg/mL) was used as a positive control of proliferation inhibition of cultured cells and complete medium was used as positive control for wound retraction in scratch assays.

#### Antiproliferative assay

Cells in 96-well plates (100 μL cells/well) were exposed to different concentrations of samples (0.25, 2.5, 25, and 250 μg/mL) in triplicate, followed by incubation for 48 h or 144 h at 37 °C and 5% CO_2_. Cells from plates without (T0 plate) or with sample addition (T1 plates) were fixed with 50% trichloroacetic acid (50 μL/well), and cell proliferation was determined by protein quantitation with sulforhodamine B at 540 nm^48–50^. The GI_50_ values (the concentration that inhibited 50% cell growth or a cytostatic effect) were determined through sigmoidal regression using Origin software (version 8.0, OriginLab Corporation, USA).

#### Scratch assay

For the scratch assay, HaCaT cells were suspended in complete medium, distributed in 12-well plates and incubated for at least 24 h at 37 °C and 5% CO_2_. After reaching confluence, the wound was performed as a straight line in each well with a sterile p200 pipet tip followed by medium removal. After washing each well with scratch medium (1 mL/well) to remove debris, cells were treated with scratch medium (untreated), treated with complete medium (5.0% of fetal bovine serum, positive control) or samples diluted in the scratch medium. The degree of wound closure was observed using a Leika reversed-phase microscope equipped with an Optikam B3 digital camera (Optika, Italy) at 0, 9, 18, and 24 h^51,52^. Samples were tested in duplicate, and two images were captured from each well. The images acquired for each sample at different times were quantitatively analyzed using ImageJ 1.8.0 analysis software, a free image-processing and analysis program. For all treatments, at 0 time, the scratched area was considered equal to 100%, and thus wound retraction was assumed to be 0%. At time points different from 0, the percentage of wound retraction was calculated according to Eq. (2).

2 $$Wound\;Retraction \left( \% \right) = \frac{{\left( {A_{0} - A_{t} } \right)}}{{A_{0} }} \times 100$$

where A_0_ is the initial wounded area obtained immediately after the scratch and A_t_ is the wound area at *t* hours (9 h, 18 h or 24 h) after the cell layer was scratched.

### Statistical analysis

Results are expressed as the mean ± standard deviation. Zetasizer software (version 8.01.4906, Malvern Panalytical) was used to acquire and analyze dynamic light scattering data. ImageJ 1.8.0 software was used to analyze the scratched area in the scratch assay quantitatively. Statistical analysis of the scratch assay was performed by mixed analysis of variance (ANOVA), in which the independent variable was the applied treatment, and the dependent variable was wound closure at three different times (9, 18, or 24 h), followed by Tukey's test comparing treatments at the same time. The data were analyzed using IBM SPSS Statistics version 24 and Origin (version 8.1.34.90, OriginLab Corporation, USA), and *p* < 0.05 was considered significant.
